# Book review: The school handbook for dual and multiple exceptionality

**DOI:** 10.3389/fpsyg.2022.1048529

**Published:** 2022-12-21

**Authors:** Christina Batrouni, Anies Al-Hroub

**Affiliations:** Department of Education, American University of Beirut, Beirut, Lebanon

**Keywords:** twice-exceptional, multiple exceptionality, gifted and talented students, intervention, school, dual exceptionalities, identification

## Summary of the book

The book (Yates and Boddison, 2020) is comprised of five chapters; dense in information regarding the over-identification of and differential intervention for dual and multiple exceptionalities (DME). The book discussed two respective topics: the collaborative strength-based model, and the person-centered approach, and detailed case studies to explain these topics.

Chapter 1 presents the importance of a multidimensional identification of dual and multiple exceptionalities (DME) due to over-identification, overlooked gender discriminants, and the need for harnessing DME individuals' economic and social potential. Chapter 2 states that the role of the teacher is a facilitator for change and not an expert, with the need for high learning potential (HLP) and special educational needs and disability (SEND) experts to be present in school environments in addition to training for the development of staff. Chapter 3 explains that successful identification and intervention require a strength-based collaborative model. The special needs coordinator and gifted (SENCO) and talented coordinator (GATCO) school collaborate to identify and intervene with school leaders to review performance and effective provision. The governor's role, in this case, would be to structure, evaluate, and hold accountable for any errors. Chapter 4 presents a person-centered co-production approach to DME. The zone of effective coproduction would come from the learner, professionals, and family. Chapter 5 explains the efficient implementation of such identifications and interventions in case studies, concluding policy reform.

## Evaluation of the content: Content and style

The book's introduction claims that the identification and intervention methods explained are easy to comprehend by anyone in the field of gifted education. Although such a statement seems strong and general at first, Chapter 3 identifies the roles of each reader at school (Yates and Boddison, 2020). There is an emphasis on UK policies on which most of the research is based, with strong contextualization. However, several case studies were explored, using both qualitative and quantitative methods, which strengthened the generalizability of the findings. Studies urged further systematic research indicating the lack of policy reform for the accommodation of dual- or twice-exceptionality (Kaufman, 2018). Unlike other books on gifted education and twice-exceptionality, this book classifies DME into four categories. It identifies them as high learning potential (HLP) with communication difficulties, HLP with cognitive and learning disabilities, HLP with social and emotional problems, and HLP with sensory or physical disabilities (Yates and Boddison, 2020). This goes beyond the strict DSM 5 categorization as seen in Kaufman (2018) and unifies such issues through a humanistic approach toward the definition itself.

The authors highlight the repeated failure of presenting a definition for DME; highlighting a definition of HLP as specific to the art and sports domains (Yates and Boddison, 2020). Such constriction builds the basis for the need for identifying giftedness overarching different domains. The authors left the door open for readers to redefine and interpret twice-exceptionality.

The book presents a neurodiverse approach to autism and other disorders by focusing on social, emotional, and educational compensation and counseling. Also, the book builds upon a comprehensive assessment; filling a gap in dyslexia identification research. Strength-based, self-interest-driven education, social and emotional support, and counseling techniques were emphasized and discussed as the lead to students' success. For such interventions to take place, initial teacher training has also been recommended with emphasis on building students' metacognitive skills (Yates and Boddison, 2020). The multi-intervention model indicates that there is a collaboration between the SENCO (Special Educational Needs Coordinator) and GATCO (Gifted and Talented Education Needs Coordinator), with further collaboration between school administrations, governors, parents, and teachers (Yates and Boddison, 2020). This collaboration allows for a positive and ethical overview of assessment and intervention and fills a gap in the literature concerning policy. This also fills a gap in the lack of evidence where SENCO and GATCO collaboration leads to student success.

## Discussion

The book offers a variety of practical teaching strategies to support SENCOs, GATCOs, educators, school leaders, and governors in developing effective provisions for gifted twice and multiple exceptional learners (Yates and Boddison, 2020). It also suggested approaches to ensure effective synergy between families and professionals.

The book introduces a comprehensive assessment in line with McCallum et al.'s (2013), Kaufman's (2018), and Al-Hroub's (2021), research. The lens that the book looks at DME through is neurodiverse, further refuting traditional testing and intervention methods to reframe within multifaceted ones. This allows for connection with previous research and prompts the reader to do so by grazing over previous policy and research errors. Through this neurodiverse lens, the book allows for a positive outlook on mental health assessment in ways that policymakers and governors must uptake. The book fills a gap in the research about evidence-based collaboration and policy by presenting six detailed cases. Such cases included quantitative assessment results and pre-post intervention through the qualitative description (Yates and Boddison, 2020). This has further implications for cross-cultural application and generalizability. Such research proved feasible for policy change in the UK and must be regarded and built on across different settings and countries.

## Author contributions

Both authors listed have made a substantial, direct, and intellectual contribution to the work and approved it for publication.
